# Targeting Autophagy for the Treatment of Alzheimer’s Disease: Challenges and Opportunities

**DOI:** 10.3389/fnmol.2019.00203

**Published:** 2019-08-22

**Authors:** Jie Liu, Lian Li

**Affiliations:** Translational Center for Stem Cell Research, Stem Cell Research Center, Tongji Hospital, Tongji University School of Medicine, Shanghai, China

**Keywords:** Alzheimer’s disease, autophagy, autophagic vesicle, amyloid-β plaque, tau neurofibrillary tangle, synapse, axon

## Abstract

Alzheimer’s disease (AD) is the most common type of dementia which characterized by a progressive loss of memory and cognitive function due to degeneration of synapses and axons. Currently, there is no cure for AD. Deposition of extracellular amyloid-β (Aβ) plaques and intracellular tau neurofibrillary tangles (NFTs) are two hallmark pathologic changes in the brains of Alzheimer’s patients. Autophagy is the major mechanism in cells responsible for removing protein aggregates. Accumulation of immature autophagic vacuoles (AVs) in dystrophic neurites of Alzheimer patients’ brains suggests that autophagy process is disrupted. Till now, it is far from clear what role autophagy plays in AD, a causative role, a protective role, or just a consequence of the disease process itself. To design more effective therapeutic strategies towards this devastating disorder, it is essential to understand the exact role of autophagy played during different stages of AD.

## Introduction

Alzheimer’s disease (AD) is a progressive neurodegenerative disorder and the most common form of dementia in the elderly, which is characterized by a progressive deficiency in memory and cognitive functions (Scheltens et al., 2016). More than 50 million people are affected by AD worldwide (Hodson, 2018). Autosomal dominant mutations in amyloid precursor protein (APP), presenilin 1 (PS1), or presenilin 2 (PS2) cause early-onset familial AD (fAD). Whereas the vast majority (>95%) of AD cases develop sporadically without a clear genetic component or etiology, which are known as sporadic AD (sAD). Additionally, as the main risk factor for AD is aging, AD is predicted to become a major socioeconomic burden in the near future with the average life expectancy on the rise (Xia et al., 2018). Deposition of extracellular amyloid-β plaques (Aβ, aggregated β-amyloid peptide) and intraneuronal tau neurofibrillary tangles (NFTs, aggregated hyperphosphorylated tau protein) in specific brain regions are two major lesions of this devastating pathology (Blennow et al., 2006). Through proteolytic cleavage by β- and γ-secretase sequentially, Aβ is generated from APP, and then secreted at the plasma membrane (Vassar et al., 1999; De Strooper et al., 2012). In aggressive early-onset fAD patients, mutations of APP or γ-secretase-associated PS1 and PS2 were identified, which strongly links Aβ to AD. The amyloid cascade hypothesis was formed based on this strong causative relationship between Aβ and AD (Hardy and Higgins, 1992; Karran et al., 2011), which assumes that the deposition of Aβ peptide in the brain is a central event in AD pathogenesis and predicts that Aβ accumulation precedes the NFTs formation. Clinically, the extent of tau NFTs formation correlates closely with the cognitive dysfunction of AD to a greater degree than does Aβ plaque load (Nelson et al., 2012). However, the etiology mechanisms underlying these pathological changes and progressive loss of synapses and neurons in AD are not clear yet.

Macroautophagy [hereafter referred to as autophagy, see review by Cuervo and Wong (2014) for chaperone-mediated autophagy] is a conserved process for clearing of long-lived proteins, protein aggregates, dysfunctional cellular organelles and invaded pathogens, which is an essential mechanism for maintenance of cellular homeostasis (Dikic and Elazar, 2018). Recently, it has become apparent that autophagy plays a central role in development, aging, and neurodegeneration. In addition, levels of autophagy are exquisitely regulated in different cells. Homeostasis and survival of neurons depend on the essential role of basal autophagy. The post-mitotic nature of neurons predisposes them to the deposition of misfolded proteins and damaged organelles which otherwise could be diluted through cell division in replicating cells (Metaxakis et al., 2018). Mice deficiency of the essential autophagy-related gene 5 (Atg5) or Atg7 specifically in the central nervous system exhibit progressive neuronal degeneration with abnormal intracellular proteins accumulated and a large number of aggregates and inclusions developed (Hara et al., 2006; Komatsu et al., 2006). Synapses are regions of quick protein turnover with high energy demand. Coordinated protein synthesis and degradation are indispensable for the morphological and functional modifications of synapses (Nikoletopoulou and Tavernarakis, 2018). It is now widely accepted that neuronal autophagy is essential for the synapse plasticity, which is required for learning and memory that is impaired in AD (Bingol and Sheng, 2011; Hernandez et al., 2012). Accumulating evidence also supports that, for the maintenance of local axon homeostasis and protection against axonal degeneration under stress conditions, normal function of autophagy is particularly important. In the distal region of the axon, Hollenbeck observed real-time autophagosome formation, which suggested autophagosome biogenesis in the axons locally (Hollenbeck, 1993). By employing live imaging, recent studies clearly showed that autophagosomes are initially produced in the distal terminals of axons, then transported to the soma retrogradely, and finally ended in completing the degradation of its contents by fusion with lysosomes (Yue, 2007; Maday et al., 2012). All of the above studies indicated that during the neurite extension and maintenance process autophagy was a key mechanism for shaping the structures of neurite and growth-cone, and was essential for neural plasticity. In the central nervous system, suppression of basal autophagy causes severe axonal swelling and atrophy, which leads to neurodegeneration eventually (Hara et al., 2006; Komatsu et al., 2006).

However, several works confirmed the scarcity of autophagosomes in healthy neurons (Mizushima et al., 2004; Nixon et al., 2005; Boland et al., 2008), which may due to high efficiency of autophagosome clearance in neurons (Boland and Nixon, 2006; Boland et al., 2008). In addition, researches show that autophagy is more efficient in young neurons than in old ones (Boland et al., 2008), as autophagy-related proteins such as Atg5, Atg7, and beclin-1 will decline with aging (Shibata et al., 2006; Lipinski et al., 2010), which probably contributes to the late onset of several neurodegenerative diseases including AD (Harris and Rubinsztein, 2011).

## Malfunction of Autophagy in AD

A substantial amount of evidence supports that autophagy dysregulation occurs in both AD patients and animal models. As early as 1967, Suzuki found that, in AD patient brains, there were a large amount of abnormal subcellular vesicles and aggregated tau protein accumulated in the swollen or dystrophic neuritis (Suzuki and Terry, 1967). The identity of these vesicles was unknown at that time. In 2005, by using immunogold labeling and electron microscopy, Nixon’s group found that these vesicles accumulated in dystrophic neurites in AD brains were immature autophagic vacuoles (AVs; Nixon et al., 2005). Data from PS1/APP double transgenic mice also showed that large amount of AVs accumulated in neuronal dendrites and soma before Aβ plaques appeared when compared to age-matched controls (Yu et al., 2005). In hippocampal neurons of AD mice, far before the synaptic and neuronal loss, abnormal accumulation of immature AVs in axon was observed (Tomiyama et al., 2010; Sanchez-Varo et al., 2012). In several other animal models of AD including TgCRND8 mice over-expressing mutant human APP695 and APP_SWE_/PS1_M146L_, the abnormal accumulation of AVs has also been observed (Cataldo et al., 2004; Yang et al., 2011a). Tau aggregates are degraded through autophagy pathway (Wang and Mandelkow, 2012; Ji et al., 2017). Autophagic gridlock also contributes to the development of AD-like tauopathy (Bakhoum et al., 2014). The abundance of AVs in the brains of AD animal models and AD patients is in sharp contrast to the rarely-observed AVs in normal brains, which suggests that the accumulation of pathogenic proteins such as Aβ and tau in AD may be caused by defective autophagy-lysosome proteolysis pathway (Cataldo et al., 2004; Yang et al., 2011a).

So far, three genes, APP, PS-1, and PS-2, have been identified as causative genes for fAD (Tang and Gershon, 2003). Research has found wild type PS1 but not mutation forms, by regulating the distribution of v-ATPase subunit V0a1 onto lysosome, is crucial for lysosome acidification and thus contributes to the regulation of autophagy-lysosome degradation system in a γ-secretase-independent way (Lee et al., 2010). As a major genetic risk factor for sAD, apolipoprotein E4 (apoE4) has been found to induce malfunction of autophagy. Aβ42 in lysosome were significantly elevated in ApoE4 transgenic mice, which finally led to neuronal death in the hippocampus (Belinson et al., 2008). In addition, other study found that in Neuro-2a cells ApoE4 potentiates leakage from lysosome and enhances Aβ peptide-induced apoptosis (Ji et al., 2006). Progressive accumulation of AVs and lysosomal deficits in the brain has been widely recognized as another hallmark of AD (Nixon and Yang, 2011), though it is still a debate whether autophagy dysfunction is the result or the cause of AD (Shin et al., 2014; Peric and Annaert, 2015). Furthermore, gender difference may have an influence on the malfunction of auto-lysosome system (Congdon, 2018). In general, accumulating evidence seems to indicate that in the early stages of AD development autophagy has a protective role, whereas it appears to potentiate neuronal degeneration in the more advanced stages.

## Mechanisms Underlying Autophagy Impairment in AD

The abnormal accumulation of autophagosomes in neurons of AD brain constituted the first sign of autophagy deficits. But, it is not fully understood what is the exact mechanism underlying dysfunction of autophagy in AD (Liang and Jia, 2014). The build-up of AVs in neurodegenerative diseases may reflect enhanced autophagy induction, impaired later lysosomal degradation steps in the autophagic pathway, or a lower rate of autophagy initiation combined with insufficient lysosome fusion and digestion (Barnett and Brewer, 2011). In general, the field is still at odds over which stage or stages of the autophagic-lysosomal pathway is dysfunctional in AD.

### Altered Autophagy Initiation in AD

Although autophagosomes are numerous in brains of the PS1_M146L_/APP_751SL_ mouse and in brains of AD patients, it does not necessarily indicate that autophagy induction is upregulated. Actually, the expression of beclin-1 which is an essential protein for autophagy initiation is decreased in brains of AD patients when compared with that of healthy individuals (Liang et al., 1999; Pickford et al., 2008). The loss of beclin-1 is believed to be caused by the increased activity of caspase 3, as this enzyme-mediated cleavage of beclin-1 occurs in brains of AD patients (Rohn et al., 2011). In an APP transgenic mouse model with beclin-1 deletion, the basal level of autophagy is disrupted, and intracellular Aβ accumulation increased (Pickford et al., 2008). Protein p62 is an autophagic cargo receptor, which was shown to bind directly to microtubule-associated protein 1A/1B-light chain 3 (LC3; Pankiv et al., 2007). In a triple transgenic mouse model of AD (3xTg-AD), Du et al. (2009a,b) observed a significant decrease in p62 expression. On the contrary, a genome-wide research indicated that autophagy is up-regulated specifically in AD, due both to the transcriptional up-regulation of positive regulators of autophagy and to the reactive oxygen species-dependent activation of a critical kinase for the initiation of autophagy, the type III PI3 kinase (Lipinski et al., 2010). Bordi et al. (2016) reported that autophagy flux increased in CA1 neurons of Alzheimer hippocampus as indicated by striking upregulation of Atgs, increases in autophagosome formation and lysosomal biogenesis beginning at early AD stages. As controversy is evident, more effort should be made in this area to assess real-time autophagy activity at different stages of AD pathogenesis. Autophagy may be regulated differentially in the early stage and late stage of AD. In this regard, developing reliable *in vivo* autophagy flux assay methodology should be a priority for the field.

### Disrupted Transportation of Autophagosome

In normal neurites, for final lysosomal degradation, immature AVs are transported retrogradely from distal axon terminals towards the soma. In the AD brain, their transportation might be impeded as suggested by the significant build-up of AVs within dystrophic neuritis (Nixon, 2007). In mouse and cell models of AD, it has also been reported that the transportation of autophagy-related compartments is selective defected (Nixon and Yang, 2011). Inhibiting the delivery of autophagosomes to lysosomes induces a rapid AVs accumulation in neurites, with very similar morphology to what has seen in the AD brain, which further suggests that defective axonal transportation of AVs may play a role in AD pathogenesis (Boland et al., 2008).

In the central nervous system, tau protein is mainly found in neurons, where it primarily localizes in axons and to a much lesser extent in dendrites and neuronal soma (Binder et al., 1985). Tau is effectively degraded through the autophagy pathway and regulates autophagy in reverse (Caballero et al., 2018). Several studies have shown that autophagy-lysosome system impairment leads to the formation of tau oligomer and insoluble aggregate, whereas their formation can be significantly alleviated through the induction of autophagy (Hamano et al., 2008; Congdon et al., 2012). In addition, autophagy dysfunction can be a result of tau hyperphosphorylation as tau modifications can provoke lysosomal aberrations (Lim et al., 2001; Lin et al., 2003). Tau is critical for autophagosome retrograde trafficking and maturation to fuse with lysosome through facilitating the assembly and stabilization of microtubule (Dixit et al., 2008). Importantly, in AD models, tau may gain a toxic function as tau deficiency is largely protective against Aβ toxicity, which suggests that Aβ-mediated neurotoxicity seems to require tau in AD (Roberson et al., 2007; Ittner et al., 2010). However, other studies offer an opposite point of view that the real causative factor of axonal dysfunction is the abnormality of lysosomal proteases (Xie et al., 2015). To clarify the molecular defects that underlie the AVs transportation failure in AD, more studies are required to identify the role of each defect.

### Defective Lysosomal Fusion/Degradation in AD

There are reports show that PS1 is critical for the acidification of lysosome and the fusion of the autophagosome with the lysosome. Abnormal accumulation of AVs have been identified in fibroblasts derived from patients with fAD-linked PS1 mutations, where markedly impairment of the turnover of long-lived proteins is detected (Esselens et al., 2004; Lee et al., 2010, 2015; Neely et al., 2011). Others believe that lysosomal calcium homeostasis defects, but not proton pump defects, causes endo-lysosomal dysfunction in PS1-deficient cells (Coen et al., 2012; Zhang et al., 2012). Anyway, supported by accumulating evidence, a critical role of lysosomal proteolytic failure has been suggested in the development of neurodegeneration in AD (Colacurcio et al., 2018). In AD brain, the AVs accumulated are electron-dense autolysosomes and autophagosomes which are filled with undigested or incompletely-digested “waste” proteins (Nixon et al., 2005). Abnormally high levels of Aβ, ubiquitinated proteins, and LC3-II are presented in the lysosome and AVs fractions isolated from the brains of TgCRND8 mice (Yang et al., 2011b). The morphology of accumulated AVs in brains of a transgenic mouse model of AD and AD patients is very similar to that induced by blockage of lysosomal proteolysis with specific cathepsin deletion (Koike et al., 2000; Boland et al., 2008). In neurons, selectively blocking of cathepsin-mediated proteolysis within autolysosomes using inhibitors of cysteine-protease or aspartyl-protease also leads to a marked accumulation of AVs with electron-dense double-membrane which contained incompletely degraded LC3-II (Boland et al., 2008). The above evidence strongly indicates the principal mechanism underlying autophagic dysfunction in AD may be the disruption of substrate proteolysis within the autolysosome.

On the other hand, works done by others suggests that APP metabolites may also accumulate in cells through inhibition of autophagy before fusion with lysosome, such as genetic deletion of ATG7 or ATG5, which indicating that AD-like pathology may arise by perturbation at any step along the autophagy-lysosome pathway (Tian et al., 2011). In addition, autophagy plays an important role in Aβ metabolism. First, autophagy is believed to be another major Aβ clearance pathway (Nilsson and Saido, 2014) along with Aβ degradation enzymes (Miners et al., 2008). Second, under physiological conditions, the autophagy-lysosome pathway is important for the degradation of Aβ; whereas under pathological condition or during the process of aging, it is demonstrated the autophagy-lysosome system is a novel pathway for the production of Aβ (Yu et al., 2005). Third, the secretion of Aβ is also mediated by autophagy. Recent findings demonstrate that extracellular release of Aβ through the autophagy pathway. Genetic deletion of essential autophagy component leads to inhibition of Aβ secretion and reduced intracellular accumulation of Aβ, which further exacerbated neurodegeneration (Nilsson et al., 2013). In return, Aβ could also regulate autophagy. In neurons, Aβ could directly induce autophagy (Hung et al., 2009), disrupt autolysosomal membrane physical integrity, and impair substrate degradation in the lysosome (Ling et al., 2009). It seems that a pro-survival role is played initially by neuronal autophagy induced by Aβ42, which is switched to a pro-death role in a time-dependent manner.

## Autophagy Modulation for AD Therapy

### Promoting Autophagy Induction

**Figure 1:**
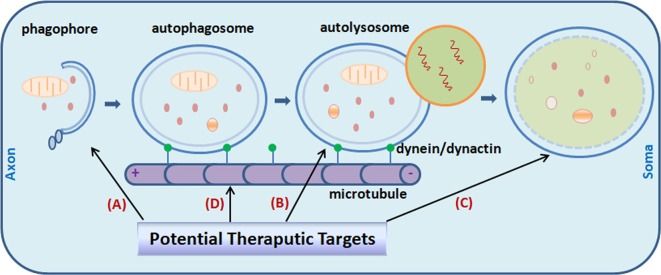
Potential targets for Alzheimer’s disease (AD) treatment through the modulating of autophagy. Promoting autophagy induction **(A)** was originally proposed as an obvious solution to reduce amyloid-β (Aβ) aggregates and tau neurofibrillary tangles (NFTs) in the AD brain. It is now realized that the efficiency is context-dependent. Regulating autophagosome-lysosome fusion **(B)** and enhancing lysosomal function **(C)** captured more attention recently. Attempts were made to stabilize retrograde transportation of the autophagosomes **(D)**. Combination therapy is also under active investigation currently. Little green dots indicate molecular motor dynein/dynactin.

By integrating many signaling cascades in the cell, the mammalian target of rapamycin (mTOR) is a well-established key pathway that senses nutrient and regulates cell metabolism (Noda and Ohsumi, 1998; Corradetti and Guan, 2006; Pei and Hugon, 2008). Genetic reduction of mTOR signaling in Tg2576 mice brain enhanced autophagy induction (Figure 1) and restored normal hippocampal gene expression signature, and results in a reduced deposit of Aβ and alleviated memory deficits (Caccamo et al., 2014). Signaling through mTOR regulates tau homeostasis (Tang et al., 2013). Pharmacologically reducing mTOR signaling with rapamycin ameliorated tau pathology (Caccamo et al., 2013). Long-term inhibition of mTOR by rapamycin or latrepirdine also prevents AD-like cognitive deficits and lowers Aβ42 level, reduces amyloid plaques and tau NFTs (McGowan et al., 2005; Caccamo et al., 2010; Spilman et al., 2010; Majumder et al., 2011). Yet it is noteworthy that the mTOR pathway itself is involved in many other critical cellular functions such as gene translation and cell growth. Toxic side-effects on patients can be induced by long-term inhibition of the mTOR pathway. So, rapamycin is not an ideal drug candidate to be considered for long-term use. Novel specific autophagy inducer is urgently needed for the field. In APP transgenic mice, administration of lentiviral vectors expressing beclin1 lead to induction of autophagy, and reduced both extracellular and intracellular amyloid pathology (Pickford et al., 2008). A point mutation (F121A) reduces the interaction between beclin1 and its endogenous inhibitor Bcl2, B-cell lymphoma 2. Knocking-in of beclin1_F121A_ in mice leads to constitutively active autophagy in multiple tissues including brain, even without any autophagy-inducing manipulation. In AD mouse models, beclin1_F121A_-mediated hyperactive autophagy significantly reduces the accumulation of amyloid, and prevents the decline of cognition, and restores the survival rate (Rocchi et al., 2017). Using a gene therapy strategy, Caccamo et al. (2017) showed that cognitive deficits in APP/PS1 mice were rescued by increasing brain p62 expression. Genetic-based approaches may provide more precisely targeted AD therapy. Stimulation of autophagy also reduces neurodegeneration in a mouse model of human tauopathy (Schaeffer et al., 2012).

On the other side, conflicting data arose from recent studies in AD models suggest that as a generalized treatment strategy for AD, the applicability of induction of autophagy is questionable. Aberrant induction of autophagy may actually lead to elevated Aβ production, as accumulated autophagic vesicles containing active γ-secretase machinery (Yu et al., 2005; Boland et al., 2008). More and more studies support the notion that the effect of autophagy modulation is context-dependent. Autophagy induction is not always beneficial. Research has shown that a major reservoir for Aβ production in AD brain may be autophagosomes (Yu et al., 2005). Induction of new autophagosome formation but not accompanied by a parallel autophagic flux increase may actually lead to increased Aβ production and catabolic contents leaking from AVs (Nixon, 2007). When considering autophagy modulation as a therapy, what is the autophagy defect, when to intervene, and how long/how strong for the modulation, all these should be taken into account. For instance, it has been reported that Aβ42-induced cell death can be alleviated through inhibition but not stimulation of autophagy (Ling et al., 2009; Wang et al., 2010). So, it appears that the benefit of enhanced induction of autophagy is context-dependent although basal autophagy is required for neuronal survival. This is further demonstrated by the findings that induction of autophagy after the formation of mature tangles and plaques had no effect on cognitive deficits or other AD-like pathology, whereas increasing autophagy induction before the development of AD-like pathology reduced the levels of soluble tau, Aβ and amyloid plaques in 3xTg-AD mice (Majumder et al., 2011). In addition, conflicting results on the role of autophagy modulation may partially arise from the differences in models. Furthermore, systematic research is necessary for detailed examining the levels of autophagic activity in different cells (neuron vs. glia) in AD as discussed below.

In addition, the accumulation of insoluble Aβ42 over time may be the direct cause of the development of autophagic dysfunction (Steele and Gandy, 2013). In support of this hypothesis, a recent report showed that no conversion to autophagic/lysosomal failure was observed when TgCRND8 mice were treated with scyllo-inositol, an endogenous inositol stereoisomer that is known to inhibit Aβ42 aggregation and fibril formation before the onset of autophagic/lysosomal failure. In contrast, immature AVs and autophagic/lysosomal substrates were significantly accumulated in vehicle-treated TgCRND8 littermates (Lai and McLaurin, 2012).

### Enhancing Lysosomal Function

Recent studies point to impaired lysosomal proteolytic function as the origin of auto-lysosome malfunction in AD pathogenesis (Yang et al., 2011b). Genetic ablation of cystatin B, an endogenous inhibitor of lysosomal cysteine proteases, in the TgCRND8 AD mouse model with clear defect in proteolytic clearance of autophagic substrates, significantly elevated the lysosomal activity (Figure 1), which leads to enhanced clearance of the autophagic substrates, and obvious alleviation of memory deficits and amyloid pathologies in the animals (Yang et al., 2011a, 2014). Pharmacological compounds with such effects would greatly facilitate research effort in this therapeutic direction (Yang et al., 2017).

### Combination Therapy

Theoretically, it would provide more benefit by simultaneously using two pharmacological autophagy-inducers that act through different regulatory pathways. Indeed, by using the mTOR-independent autophagy enhancer trehalose or lithium and the mTOR-dependent autophagy enhancer rapamycin in combination upregulates autophagy to a greater extent and leads to quicker clearing of protein aggregates than using each alone (Sarkar et al., 2005, 2008). Moreover, using two drugs in combination may enable reduction of the dose of each treatment when compared with either treatment alone, which might greatly reduce the likelihood of adverse effects. In such a scenario, it might be a promising intervention strategy to moderately increase autophagy induction in combination with methods to promote the successful completion of autophagic degradation. However, it is still a big challenge to target the defective lysosomal proteolysis and the autophagy induction at the same time.

In this regard, as it coordinately activates lysosomal biogenesis as well as genes required for autophagosome formation, it seems that transcription factor EB (TFEB) fulfills both of these criteria (Cortes and La Spada, 2019). With its efficacy has already been showed under several neurological conditions, including lysosomal storage disorders (Song et al., 2013), Huntington’s disease (HD; Vodicka et al., 2016) and Parkinson’s disease (PD; Decressac et al., 2013), it is expected that in the AD context similar benefits may also be achieved. Actually, a study published recently provides the first evidence that TFEB may indeed be beneficial for AD treatment (Xiao et al., 2015). On the other hand, pharmacological treatments which improve the catalytic performance of lysosomal enzymes and simultaneously reducing the burden of auto-lysosomal pathway would be another way to tackle the problem of AVs clearance slowdown and disturbed lysosomal function, as exampled by the study of Yang et al. (2017; Figure 1).

### Other Strategies

Alternatively, interventions aiming at reducing the burden to the inappropriate functioning autolysosomal compartments hold some potential as well. For example, preventing Aβ production and oligomerization along with lowering cholesterol may all prove beneficial. In an AD model, it has been demonstrated that 2-hydroxypropyl-beta-cyclodextrin, a cholesterol-lowering drug, is indeed emerging as a potential useful pharmacological tool (Ren et al., 2016). As for Aβ, a recent work implies that useful selective inhibitors of Aβ production could be developed from peptides that disrupt the physical interaction between the APP and PS1 (Das et al., 2003). Finally, as growing evidence suggests that restoring proper endosomal trafficking (recycling) may have a similar effect, another potential strategy to tackle this issue is to develop specific pharmacological modulators of these processes. Two recent studies provided the first proof of concept test through developing pharmacological stabilizer of the retromer sorting complex for AD treatment (Mecozzi et al., 2014; Young et al., 2018).

## Disturbed Autophagy in Glial Cells in AD

Astrocytes, microglia, and oligodendrocytes are important components involved in the AD pathogenesis (Dzamba et al., 2016). Under normal conditions, they perform supporting and surveillance functions to neurons in the central nervous system. Engulfing and phagocytosis of extracellular “garbage” like Aβ is vital for neuronal homeostasis. Studies showed that autophagy in glial cells also played a key role in cleaning the microenvironments around neurons (Xue et al., 2014; Pomilio et al., 2016). Disturbing basal autophagy process in glia leads to gliosis and neuroinflammation, which contribute significantly to the development and progression of AD (Herrup et al., 2013; She et al., 2018). Autophagy in glial cells should be taken into account when targeting this process for the treatment of AD.

## Conclusions and Future Directions

The auto-lysosomal function is clearly impaired in AD, which contributes to the accumulation of Aβ plaques and tau NFTs, the two most significant hallmarks of AD. Specific diagnostic methods are urgently needed for accurately identifying and quantifying the autophagic dysfunction *in vivo* in AD. Currently, the “gold standard” for monitoring autophagy in tissue is direct observation under transmission electron microscopy. It has also been widely used to assess autophagy by Immunohistochemical staining and immunoblotting against autophagy-specific biomarkers such as LC3 (Klionsky et al., 2016). Other approaches include forced expression of GFP-LC3 to detect AVs under the microscope as fluorescent dots, and the use of weakly basic dyes which accumulate in the acidic autophagosome-lysosome compartments (Klionsky et al., 2016). Till now, there is no method to monitor autophagy activity *in vivo* in a real-time manner.

For a deeper understanding of the dysfunction of autophagic in AD and for the successful development of therapeutic strategies based on autophagy modulation, it is also very critical to exploring biomarkers that can be applied widely in clinical settings to assess the therapeutic efficiency of autophagy modulation (Chiong, 2018). In addition, as evidence against the druggability of autophagy pathway in the late-stage of the disease, more studies should aim to consider preventive or intervention trials in the early stage of AD (Steele et al., 2013).

## Author Contributions

LL conceptualized and designed the study. LL and JL reviewed the literature, wrote the manuscript, and drew the figure; read and approved the final manuscript.

## Conflict of Interest Statement

The authors declare that the research was conducted in the absence of any commercial or financial relationships that could be construed as a potential conflict of interest.

## Abbreviations

AD: Alzheimer’s disease
APP: amyloid precursor protein
PS1: presenilin 1
PS2: presenilin 2
fAD: familial AD
sAD: sporadic AD
Aβ: amyloid-β
NFTs: neurofibrillary tangles
Atg: autophagy-related genes
AVs: autophagic vacuoles
apoE4: apolipoprotein E4
LC3: microtubule-associated protein 1A/1B-light chain 3
mTOR: mammalian target of rapamycin
Bcl2: B-cell lymphoma 2
TFEB: transcription factor EB
HD: Huntington’s disease
PD: Parkinson’s disease.
